# IGF-1 and GLP-1 signaling: potential therapeutic target for neurological disorders – correspondence

**DOI:** 10.1097/MS9.0000000000000164

**Published:** 2023-01-23

**Authors:** Neeraj Gajwani, Kamna Rawat, Akshita Sharma, Mahesh Rachamalla, Niraj K. Jha, Hitesh Chopra, Kuldeep Dhama, Talha B. Emran

**Affiliations:** a AMA School of Medicine, Makati, Philippines; b Government Doon Medical College; c Department of Biotechnology, School of Applied and Life Sciences (SALS), Uttaranchal University, Dehradun, Uttarakhand; d Veer Chandra Singh Garhwali Government Institute of Medical Science and Research, Srikot, Srinagar; e Department of Biotechnology Engineering and Food Technology, Chandigarh University, Mohali; f Chitkara College of Pharmacy, Chitkara University, Chandigarh, Punjab; g Department of Biotechnology, School of Engineering and Technology (SET), Sharda University; h Division of Pathology, ICAR-Indian Veterinary Research Institute, Bareilly, Izatnagar, Uttar Pradesh, India; i Department of Biology, University of Saskatchewan, Saskatoon, Saskatchewan, Canada; j Department of Pharmacy, BGC Trust University Bangladesh, Chittagong; k Department of Pharmacy, Faculty of Allied Health Sciences, Daffodil International University, Dhaka, Bangladesh

Neuronal cell survival, proliferation, and neurogenesis are all dependent on insulin-like growth factor-1/glucagon like peptide-1 (IGF-1/GLP-1) signaling. In the last few decades, the downregulation of IGF-1 and GLP-1 has been linked to problems in the nervous system and these two proteins have become possible therapeutic targets for a number of neurodegenerative and neuropsychiatric disorders. Consequentially, activation of the IGF-1/GLP-1 receptor may play a vital role in neuronal protection. In their review, Bhalla *et al*.^1^ put together evidence about how IGF-1 and GLP-1 signaling target activators could be used to treat neurological disorders. The analogy of IGF-1 and GLP-1 cross the blood–brain barrier and perform neuroprotective functions such as synaptic formation, neuronal plasticity, protein synthesis, and autophagy. A growing body of literature suggests that disturbance of these pathways contributes to the progressive loss of neurons in the two most common neurodegenerative disorders, Alzheimer’s disease (AD) and Parkinson’s disease (PD). These findings have prompted numerous studies in preclinical models of neurodegenerative disorders using currently available antidiabetics to target insulin, IGF-1, and GLP-1 signaling. These studies indicate that insulin, IGF-1, and GLP-1 agonists fix signaling problems and improve surrogate markers of neurodegeneration as well as behavioral outcomes. Several proof-of-concept studies are currently underway to try to apply promising preclinical results to patients suffering from AD and PD^2^.

IGF-I and IGF-II are peptide mitogens expressed in various tissues including the nervous tissues. These polypeptides as well as their receptors are found abundantly in the hypothalamus, thalamus, hippocampus, and cortex. IGF exerts its effect on the cells through a transmembrane receptor. The downstream action of the molecule is similar to insulin through tyrosine kinase. The IGF protein family has an important role in the growth, development, and prevention of oxidative damage to the nervous system. IGF-binding proteins are proteins that bind IGF to regulate their levels in the tissue. IGF proteins are also implicated in optimal neurocognitive function. Decreased function of IGF may play a role in the disruption of its physiological neuroprotective actions and may cause deleterious sequelae like demyelination, oligodendrocyte function, and neuroinflammation. IGF may exert its neuroprotective effect by regulating glucose metabolism and enhancing mitochondrial function in glial cells. Preclinical and clinical studies have shown that IGF may have a pharmacological role in treating dementia disorders like AD, PD, and Huntington’s disease attributed to the formerly described mechanisms. Despite limited trials, the evidence of its usefulness as a neuroprotective agent in all these disorders is undeniable^1^,^3^.

Inflammation, oxidative stress, and apoptosis have all been linked to PD etiology. There is some evidence that insulin resistance arises in PD, however, this is debatable because systemic insulin resistance is not present in de novo, medication-free PD patients, and brain insulin resistance is not present in cognitively normal PD cases. GLP-1 promotes PD pathogenesis via its G protein-coupled receptor which activates both cAMP-PKA and PI3K pathways via Akt, PKC, and MAPK. GLP-1 is synthesized by neurons of the brainstem’s solitary tract. Furthermore, when produced from the gut, this peptide stimulates the GLP-1 receptor found on vagal sensory neurons that comprise the hepatoportal glucose sensor, connecting with brainstem neurons and extending its activity to multiple brain areas. GLP-1 has been proven in several studies to alter neuronal functions such as thermogenesis, blood pressure management, neurogenesis, neurodegeneration, retinal healing, and energy homeostasis^4^. Since GLP-1 signaling appears to have favorable effects on various parameters implicated in neurodegenerative disorders such as endoplasmic reticulum stress, poor redox homeostasis, autophagy, and chronic inflammation, therefore, presenting GLP-1 receptor agonists (GLP-1RAs) as intriguing therapeutic candidates for neuronal loss and neuroinflammation. Recombinant GLP-1 has been proven in numerous experimental trials to alleviate neurological impairment and reduce brain infarct area, primarily by inhibiting oxidative stress and apoptosis^5^. Also, their neuroprotective significance in AD and PD has been recognized. Despite the enormous body of evidence supporting the neuroprotective effects of GLP-1RAs in animal models of AD human trials are still lacking, or the results that have been presented have been inconclusive, warranting more conclusive human evidence^6^. The Food and Drug Administration has previously approved many GLP-1RAs for the treatment of diabetes and obesity, and they could be repurposed for the treatment of neuroinflammation and neurodegeneration. More studies in human clinical trials will be required to confirm the safety, tolerability, and efficacy of each medicine in lowering neuroinflammation in the human brain as well as show the implications for neurodegenerative disease treatment. Several clinical trials to investigate the utility of GLP-1RAs in treating neurodegenerative diseases have been completed or are currently underway, and the use of exosomes to evaluate biomarkers of drug-target engagement and biological cascades involved in disease progression would provide valuable insight into drug action^7^.

The authors missed covering the prospect of triple receptor agonists [GLP-1, glucose-dependent insulinotropic polypeptide (GIP), and glucagon receptors agonist]. Recently triple receptor agonists which stimulate GLP-1, GIP, and glucagon receptors have been developed. Pathophysiological improvements in AD mice models like antiapoptotic effects, reduced amyloid β deposition and phosphorylated hippocampal tau, protection from synapse loss, reduced inflammatory and oxidative stress responses in the cortex and hippocampus, and elevated brain-derived neurotrophic factor. Furthermore, synaptophysin and postsynaptic density protein-95, which reflect synaptic damage, have been seen to be increased while neuronal excitability was restored and intracellular calcium, a crucial regulator in metabolic dysfunction and progressive neuronal death, was regulated. Long-term memory impairment in spatial maze tests was also improved by treatment. Despite their initial promise, it is uncertain whether the favorable benefits of triple agonists differ from those reported with dual GLP-1/GIP agonists. In comparison to single GLP-1RAs, both triple and dual receptor agonists significantly increase cAMP generation. However, triple agonists have recently been shown to have a stronger effect with triple agonists giving a larger neuroprotective advantage against glutamate excitotoxicity when compared to dual receptor agonists^8^.

The authors’ article provides a good understanding of the protective role of IGF-1 and GLP-1 signaling activation in neurological dysfunctions, but it appears to highlight a number of contradictory and ambiguous conclusions that cannot be easily generalized. These drugs show potential, but their repurposing and applicability require more human/clinical trials and comparison against other combined agonists.

## Ethical approval

Not applicable.

## Patient consent

Not applicable.

## Source of funding

None.

## Author contribution

N.G.: conceptualization, data curation, writing – original draft preparation, and writing – reviewing and editing. K.R., A.S., M.R., N.K.J., H.C., and K.D.: data curation, writing – original draft preparation and writing – reviewing and editing. T.B.E.: writing – reviewing and editing, visualization, and supervision.

## Conflicts of interest disclosure

The authors declare that they have no financial conflict of interest with regard to the content of this report.

## Research registration unique identifying number (UIN)

None.

## Guarantor

Talha B. Emran, PhD, Associate Professor, Department of Pharmacy, BGC Trust University Bangladesh, Chittagong 4381, Bangladesh. Tel: +880 303 356 193, fax: +880 312 550 224. https://orcid.org/0000-0003-3188-2272. E-mail: talhabmb@bgctub.ac.bd

## Declaration of competing interest

We have read and understood the policy on declaration of interests and have no relevant interests to declare. The responsibility for the content lies with the author and the views stated herein should not be taken to represent those of any organizations or groups with and for which he works.

## Provenance and peer review

Not commissioned, externally peer-reviewed.
